# SOCIAL CHARACTERISTICS OF THE FOURTH AGE

**DOI:** 10.1093/geroni/igz038.2564

**Published:** 2019-11-08

**Authors:** John Pothen, Emily C Dore, Ellen Idler

**Affiliations:** Emory University, Atlanta, Georgia, United States

## Abstract

How can we differentiate distinct phases of aging in later life? Theorizations of the third and fourth age posit that later life often involves a time of continued growth and increased opportunity (the “third age”) as well as a time marked by growing cognitive, physical, and social losses (the “fourth age”). In contrast to population-based definitions that place this transition around the age of 80, a person-based definition using frailty as a marker offers more sensitivity by focusing on ability and agency instead of age alone. In this study, we apply both definitions in order to examine the social characteristics of the fourth age. Using a nationally representative sample of adults over the age of 65 from from the National Health and Aging Trends Study (NHATS) seventh round (n=6,312) we find that the population-based definition overestimates the number of adults in the fourth age (2,834 vs 569; p<0.001). Additionally, social network patterns observed when comparing adults above and below the age of 80 - increased rates of including a daughter or son and a decreased rate of including a friend - are not seen when comparing adults who do and do not meet criteria for frailty. Our findings suggest that common understandings of the social characteristics of the oldest old - understandings with important implications for policy and the promotion of human dignity - may be biased by focusing on age alone as a marker of change instead of ability and agency.

